# Antimicrobial resistance among uropathogens in the Asia-Pacific region: a systematic review

**DOI:** 10.1093/jacamr/dlab003

**Published:** 2021-02-27

**Authors:** Adhi Kristianto Sugianli, Franciscus Ginting, Ida Parwati, Menno D de Jong, Frank van Leth, Constance Schultsz

**Affiliations:** 1 Department of Clinical Pathology, Faculty of Medicine, Universitas Padjadjaran, Hasan Sadikin General Hospital, Bandung, Indonesia; 2 Department of Internal Medicine, Faculty of Medicine, Universitas Sumatera Utara, Adam Malik General Hospital, Medan, Indonesia; 3 Department of Medical Microbiology, Amsterdam University Medical Centers, University of Amsterdam, Amsterdam, The Netherlands; 4 Department of Global Health, Amsterdam University Medical Centers, University of Amsterdam, Amsterdam, The Netherlands; 5 Amsterdam Institute for Global Health and Development, Amsterdam, The Netherlands

## Abstract

**Background:**

Antimicrobial resistance (AMR) in urinary tract infections (UTI) is a global public health problem. However, estimates of the prevalence of AMR, required for empirical treatment guidelines, are lacking for many regions.

**Objectives:**

To perform a systematic review and summarize the available information about AMR prevalence among urinary *Escherichia coli* and *Klebsiella pneumoniae*, the two priority uropathogens, in the Asia-Pacific region (APAC).

**Methods:**

PubMed, EBSCO and Web of Science databases were searched for articles (2008–20), following PRISMA guidelines. The prevalence of resistance was calculated and reported as point estimate with 95% CI for antimicrobial drugs recommended in WHO treatment guidelines. Data were stratified by country and surveillance approach (laboratory- or population-based surveillance). The quality of included articles was assessed using a modified Newcastle-Ottawa Quality Assessment Scale.

**Results:**

Out of 2400 identified articles, 24 studies, reporting on 11 (26.8%) of the 41 APAC countries, met the inclusion criteria. Prevalence of resistance against trimethoprim/sulfamethoxazole, ciprofloxacin, and ceftriaxone ranged between 33% and 90%, with highest prevalence reported from Bangladesh, India, Sri Lanka and Indonesia. Resistance against nitrofurantoin ranged between 2.7% and 31.4%. Two studies reported data on fosfomycin resistance (1.8% and 1.7%). Quality of reporting was moderate.

**Conclusions:**

We show very high prevalence estimates of AMR against antibiotics commonly used for the empirical treatment of UTI, in the limited number of countries in the APAC for which data are available. Novel feasible and affordable approaches that facilitate population-based AMR surveillance are needed to increase knowledge on AMR prevalence across the region.

## Introduction

Antimicrobial resistance (AMR) is a global public health threat.^1^^,^^2^ Most of the direct and indirect burden of AMR is anticipated in low- and middle-income countries due to several factors, including lack of surveillance capacity and systematic data collection of AMR.^3^^,^^4^ The Asia-Pacific region (APAC), which comprises the South-East Asia and Western Pacific Regions, is considered at high risk and a hotspot for the spread of AMR.^4^ The AMR in APAC affects both the low-to-middle-income countries and high-income countries in this region.

Urinary tract infections (UTIs) are common bacterial infections that occur both in the community and in hospitals. UTIs are mostly treated empirically and lead the rising prevalence of AMR.^5^^,^^6^ The effectiveness of empirical treatment is dependent on the underlying prevalence of resistance in the most common causative pathogens, which is often unknown due to lack of diagnostics, or is based on laboratory-based surveillance data only.^5^^,^^6^ We have previously shown in a population-based surveillance in Indonesia that the prevalence of AMR in the main urinary pathogens *Escherichia coli* and *Klebsiella pneumoniae* is extremely high (more than 50%) in both in-patients and out-patients, with a prevalence of resistance <20% only for tigecycline and fosfomycin.^7^ These results raise concerns about the prevalence of AMR in urinary pathogens in other countries in the region. Given the current unavailability of published national surveillance data for most countries, the aim of this systematic review is to summarize the available information about AMR prevalence among *E. coli* and *K. pneumoniae* isolated from patients suspected of UTI in the APAC, to inform clinical practice with regard to empirical treatment and to identify the main knowledge gaps. We focused our review on the combination of *E. coli* and *K. pneumoniae* given the fact that these two pathogens together cause up to 80% of UTIs, and as per WHO recommendations.^1^

## Methods

### Search strategy

We searched PubMed, EBSCO, and Web of Science in the time period between 1 January 8 and 5 January 2020. The search strategy included the combination of the following keywords with free text search category: ‘antimicrobial resistance’, ‘surveillance’, ‘survey’, ‘prevalence’, ‘epidemiology’ and ‘UTI’. The detailed search strategy is provided in Method 1 (available as Supplementary data at *JAC-AMR* Online). Our search strategy did not include limits for countries of the Asia-Pacific region but instead, this selection was made as part of the inclusion process.

### Selection criteria

Articles were screened by title and abstract to select those for full text assessment. Studies were included for analysis if they fulfilled the following criteria:

1. Describe AMR in bacterial isolates from human patients with symptomatic UTI (upper or lower).
2. Report on patients in the Asia-Pacific region as defined by the United Nations (Australia, Bangladesh, Bhutan, Brunei, Cambodia, China, Hong Kong, Taiwan, Macau, Cook Islands, Democratic People’s Republic of Korea, Fiji, India, Indonesia, Japan, Kiribati, Laos, Malaysia, Maldives, Marshall Islands, Micronesia, Mongolia, Myanmar, Nauru, Nepal, New Zealand, Niue, Palau, Papua New Guinea, Philippines, Republic of Korea, Samoa, Singapore, Solomon Islands, Sri Lanka, Thailand, Timor-Leste, Tonga, Tuvalu, Vanuatu and Vietnam).
3. Describe the surveillance approach (population- and/or laboratory-based).
4. Describe the microbiology procedures for identification and susceptibility testing.
5. Report the percentage of resistance to an antimicrobial drug with information on total number of isolates tested.
6. Published in English language.

Studies were excluded if they (1) reported on outbreaks, (2) reported on isolates obtained from animals, environment or food, (3) reported a systematic review, case series or a case report, or (4) lacked information regarding clinical suspicion of UTI at isolate level.

### Selection procedure

Two authors (A.K.S., F.G.) screened the title and abstract of all the articles identified through the search strategy independently. Any discrepancies during the screening process were resolved through consensus between the two assessors. Full-text of articles was assessed by a single author (A.K.S.), but two additional authors (C.S., F.vL.) were consulted to reach consensus when uncertainty on inclusion arose.

### Data extraction

Data extraction was done using a predesigned data collection tool, developed for the purpose of this review, and information stored in Microsoft Excel 2017. Data extracted included article information (first author, year of publication, time period of data collection, and country where surveillance was carried out), study design (population-based and/or laboratory-based, retrospective or prospective, sample size, age group, number of specimens collected), pathogen identification method (*E. coli* and *K. pneumoniae*) and antimicrobial susceptibility test (AST) methodology (instrument, guideline used for breakpoints, and quality control), and antimicrobial resistance data. Resistance data included total number of isolates obtained, number of isolates tested, and the number of resistant strains per bacterial species (*E. coli* and *K. pneumoniae)*. We followed WHO GLASS for listing of antimicrobial drugs.^8^ The antimicrobial susceptibility test results were collected for co-trimoxazole (SXT), ciprofloxacin (CIP), levofloxacin (LVX), ceftriaxone (CRO), cefotaxime (CTX), ceftazidime (CAZ), cefepime (FEP), imipenem (IPM), meropenem (MEM), ertapenem (ETP), doripenem (DOR), colistin (CT), fosfomycin (FOS), and nitrofurantoin (NIT), as susceptible, intermediate or resistant, as reported in the original article. For studies reporting resistance prevalence data for multiple populations, e.g. outpatients and inpatients, data were extracted for each study population; this implied having a larger number of prevalence data than the number of studies included.

### Quality assessment

The quality of each article was assessed using a Newcastle-Ottawa Quality Assessment Scale, modified for the purposes of this study (Supplementary data, Methods 2).^9^ We assessed the following quality criteria: (1) definition of study population; (2) representativeness of the sample; and (3) ascertainment of AST method.

### Data analysis

All extracted data were used directly, or recalculated as a prevalence of resistance for each antimicrobial drug assessed. Intermediate susceptibility test results were classified as resistant. The prevalence of resistance is reported as a point estimate with its associated 95% CI. Given the aim of the study to inform empirical treatment decisions, a combined analysis for *E. coli* and *K. pneumoniae* was done for each study. However, the number of *E. coli* and *K. pneumoniae* isolates contributing to each study was reported to assess the relative contribution of each species to the overall data. Data are reported stratified by surveillance strategy, i.e. laboratory-based surveillance (LBS) and population-based surveillance (PBS). We defined a study as population-based if the reported data were obtained from a defined population with signs and symptoms indicating suspicion of UTI. A study was defined as laboratory-based if data were obtained as part of routine diagnostic laboratory procedures of urine samples submitted to the laboratory because of a clinical suspicion of UTI.

Studies that provided aggregated data from multiple countries are reported separately. We did not conduct a meta-analysis because of the small number of studies available per country, and the anticipated large variation in study protocols between countries. Statistical analyses and visualization of reported data and graph were performed using STATA v12 (STATA, TX, USA).

## Results

**Figure 1.**
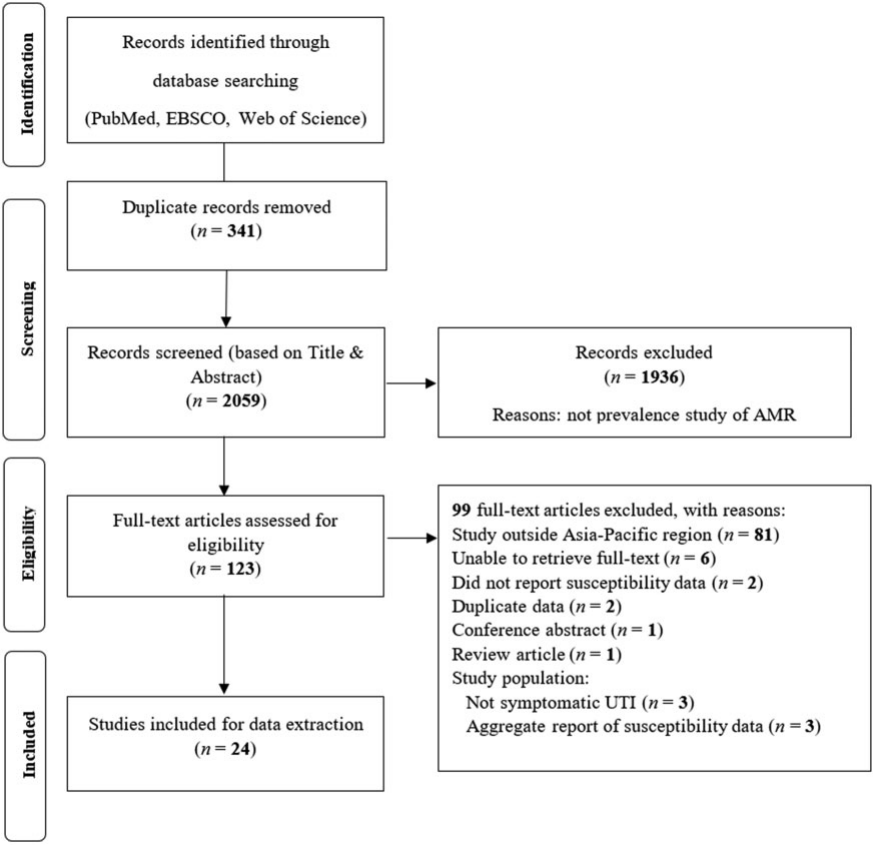
Flow chart of the systematic review process (PRISMA).

In total, 2400 articles were identified of which 24 studies^10–33^ met the inclusion criteria and were included in the final analysis (Figure 1). Data are reported for 12 population-based surveillance studies and 12 laboratory-based surveillance studies which originated from 11 countries. Studies were carried out between 2008 and 2019 and included between 47 and 7491 isolates that were studied prospectively (21 studies) or retrospectively (3 studies). Nine studies only reported data for *E. coli* and not for *K. pneumoniae* (Table S1). Studies described AMR data in a range of patient populations, including in- and out-patients, patients with and without urinary catheters, and patients with lower and upper UTIs (Table S1). Three articles reported on aggregated data for multiple countries (Table S4).

### Quality assessment

Nineteen out of 24 (79%) articles described the inclusion criteria of the study conducted. Fourteen of 24 (58%) articles described the sampling procedure, whilst 13 (54%) reported the microorganism identification procedure. Almost all articles (22/24; 92%) described the laboratory guidelines for the procedure of AST and the breakpoints used, but the quality control procedure was only reported in 10 (42%) of the articles (Table 1). Studies reported percentage of isolates susceptible or resistant, and only five studies reported on intermediate test results (Tables S2 and S3).

**Table 1.** Quality assessment of included articles

Author	Reference	Year	Definition of population		Representativeness of the sample	Ascertainment of AST method		
			Is the study population clearly described?	Are the criteria (case definition) for enrolment in the study clearly stated?	Is the sampling of the study population clearly described?	Does the study describe the method for susceptibility testing used?	Did the study specify the breakpoint standard used?	Did the study report on internal quality control measures?
Kothari	10	2008	+	+	**−**	+	+	+
Kim	11	2008	+	+	+	+	+	−
Ho	12	2010	+	+	+	+	+	+
Lee SJ	13	2011	+	+	+	+	+	−
Lu PL	14	2012	+	**−**	**−**	+	+	+
Lee DS	15	2013	+	+	+	+	+	−
Chen	16	2013	+	+	+	+	+	−
Mitchell	17	2014	+	**−**	**−**	+	+	+
Niranjan	18	2014	+	+	**−**	+	+	−
Kapur	19	2014	+	+	+	+	+	−
Hossain	20	2014	+	**−**	**−**	+	+	−
Senadheera	21	2016	+	+	+	+	+	−
Fasugba	22	2016	+	+	+	+	+	+
Amornchai charoensuk	23	2016	+	+	+	−	−	−
Jean	24	2016	+	+	**−**	+	+	+
Adeep	25	2016	+	+	+	+	+	+
Mishra	26	2016	+	+	−	+	+	−
Pruetpongpun	27	2017	+	+	+	+	+	−
Fernando	28	2017	+	+	−	+	+	−
Sugianli	29	2017	+	+	+	+	+	+
Veeraraghavan	30	2018	+	−	−	+	+	+
Choe HS	31	2018	+	−	+	−	+	−
Lee H	32	2018	+	+	−	+	+	−
Ganesh	33	2019	+	+	+	+	+	+

Abbreviations: (+), yes; (−), no.

### Prevalence of AMR

**Figure 2.**
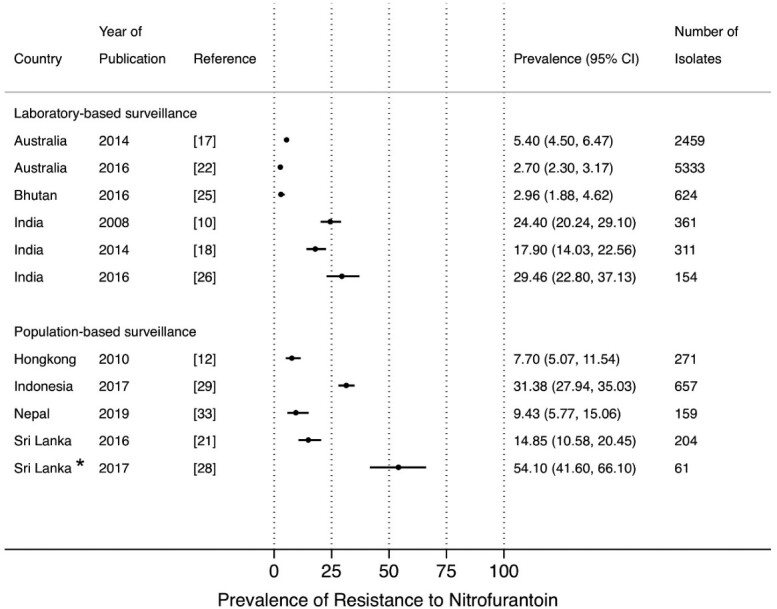
Point prevalence estimates of resistance to nitrofurantoin in *E. coli* and *K. pneumoniae* by country in the Asia-Pacific region, stratified by surveillance strategy (laboratory-based surveillance, population-based surveillance). The horizontal line indicates the 95% CI. An asterisk (*) indicates the study only included ESBL-producing isolates.

**Figure 3.**
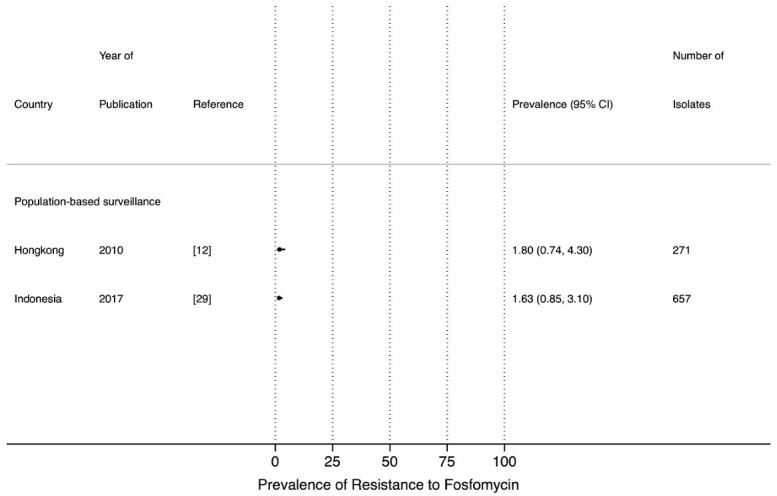
Point prevalence estimates of resistance to fosfomycin in *E. coli* by country in the Asia-Pacific region, stratified by surveillance strategy (laboratory-based surveillance, population-based surveillance). The horizontal line indicates the 95% CI.

The most frequently reported susceptibility data were for co-trimoxazole, ciprofloxacin, ceftriaxone and nitrofurantoin, as the most commonly used antimicrobial drugs to treat UTI. No studies reported data for doripenem or colistin. Nitrofurantoin and fosfomycin are considered the preferred oral antimicrobial drugs in several guidelines for UTI treatment.^34^^,^^35^ The prevalence of resistance to nitrofurantoin from PBS^12^^,^^21^^,^^28^^,^^29^^,^^33^ and LBS,^10^^,^^17^^,^^18^^,^^22^^,^^25^^,^^26^ ranged between 7.7% and 31.4%, and 2.7% and 29.5%, respectively, except for a study from Sri Lanka in 2017 which showed a high prevalence of resistance to nitrofurantoin (54.1%) (Figure 2). The prevalence of resistance to fosfomycin in *E. coli* was reported in only two studies, one each from Indonesia (1.63%) and Hong Kong (1.8%) (Figure 3).^12^^,^^29^

**Figure 4.**
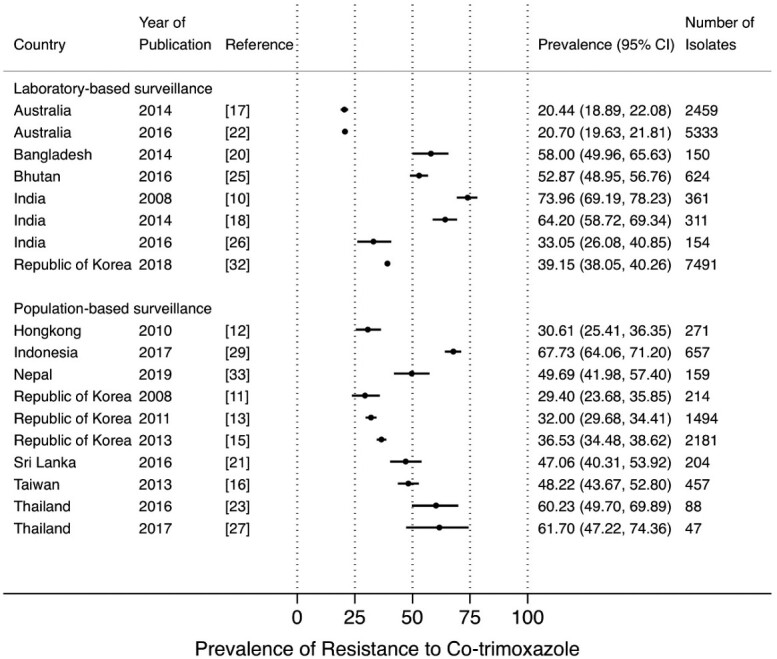
Point prevalence estimates of resistance to co-trimoxazole in *E. coli* and *K. pneumoniae* by country in the Asia-Pacific region, stratified by surveillance strategy (laboratory-based surveillance, population-based surveillance). The horizontal line indicates the 95% CI.

The prevalence of resistance to co-trimoxazole ranged between 20.4% and 73.9% in LBS^10^^,^^17^^,^^18^^,^^20^^,^^22^^,^^25^^,^^26^^,^^32^ and between 29.4% and 67.7% in PBS.^11–13^^,^^15^^,^^16^^,^^21^^,^^23^^,^^27^^,^^29^^,^^33^ A high prevalence of resistance to co-trimoxazole from LBS was observed in studies from Bangladesh (58.0%), Bhutan (52.9%), and India (between 64.2% and 73.9%). High prevalence of resistance to co-trimoxazole from PBS was reported in studies from Indonesia (67.7%) and Thailand (between 60.2% and 61.7%) (Figure 4).

The prevalence of resistance to ciprofloxacin from LBS^10^^,^^17–20^^,^^22^^,^^30^^,^^32^ and PBS^11–13^^,^^15^^,^^21^^,^^23^^,^^28^^,^^29^ ranged between 6.3% and 79.6%, and 12.9% and 90.1%, respectively, with high prevalence in Bangladesh, India, Sri Lanka, and Indonesia (Figure 5a). Five studies reported resistance to levofloxacin in both LBS and PBS which ranged between 34.8% and 71.3%, and 15.2% and 71.5%, respectively (Figure 5b).^13^^,^^16^^,^^26^^,^^29^^,^^30^

**Figure 5.**
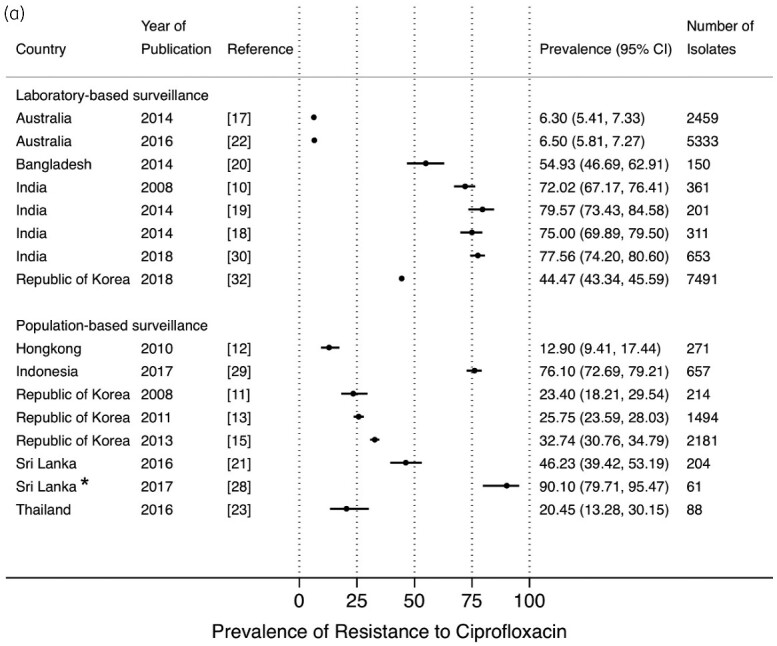
Point prevalence estimates of resistance to fluoroquinolones in *E. coli* and *K. pneumoniae* by country in the Asia-Pacific region, stratified by surveillance strategy (laboratory-based surveillance, population-based surveillance): (a) ciprofloxacin; (b) levofloxacin. The horizontal line indicates the 95% CI. An asterisk (*) indicates the study only included ESBL-producing isolates.

**Figure 5.**
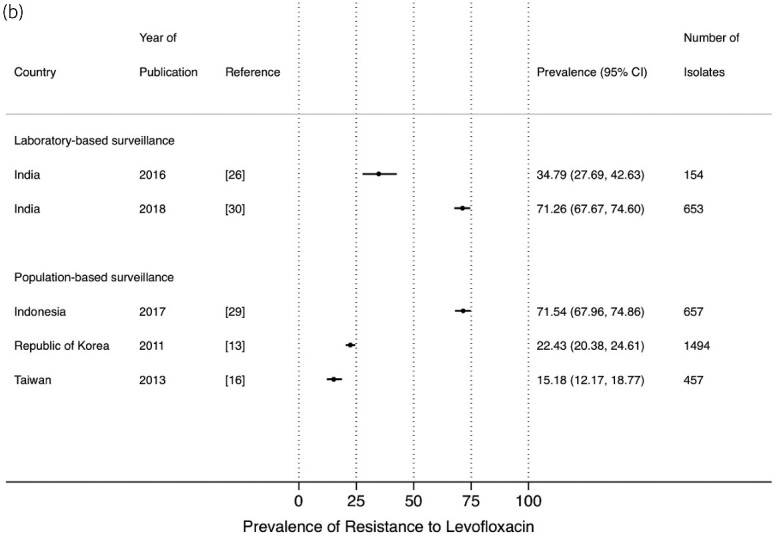
Continued.

The prevalence of resistance to ceftriaxone and cefotaxime from LBS^17^^,^^20^^,^^25^^,^^26^^,^^30^^,^^32^ and PBS,^13^^,^^15^^,^^16^^,^^23^^,^^27^^,^^29^^,^^33^ ranged between 4.3% and 74.7%, and 7.1% and 72.3%, respectively (Figure 6a and b). High resistance prevalence estimates of ceftriaxone and cefotaxime were observed in studies from Bangladesh (61.7%), India (75.1%) and in Indonesia (72.3%). A similar wide range of point prevalence estimates was observed for the other commonly used third-generation cephalosporin, ceftazidime (Figure 6c).^15^^,^^17^^,^^20^^,^^23^^,^^26^^,^^29^^,^^30^^,^^32^

**Figure 6.**
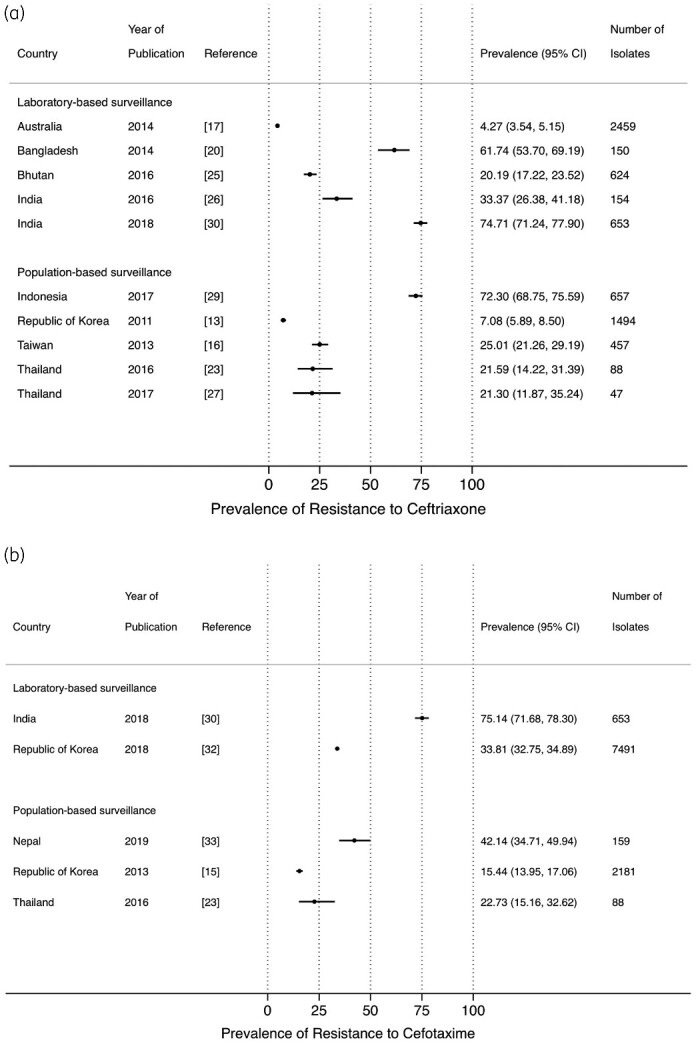
Point prevalence estimates of resistance against cephalosporins in *E. coli* and *K. pneumoniae* by country in the Asia-Pacific region, stratified by surveillance strategy (laboratory-based surveillance, population-based surveillance): (a) ceftriaxone; (b) cefotaxime; (c) ceftazidime. The horizontal line indicates the 95% CI.

**Figure 6.**
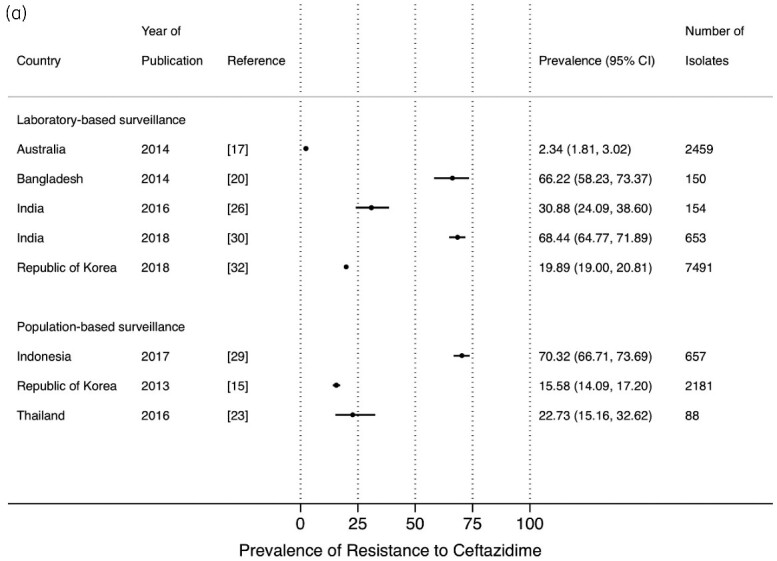
Continued.

Studies that reported on the prevalence of resistance to carbapenems were limited, in both PBS^15^^,^^16^^,^^28^^,^^29^ and LBS^18^^,^^20^^,^^30^^,^^32^ (Figure 7). Prevalence of resistance to imipenem, ertapenem and meropenem from PBS reached up to 25% in a study from Sri Lanka. A high prevalence of resistance to meropenem (51.7%) from LBS was observed in a study from Bangladesh (Figure 7c).

**Figure 7.**
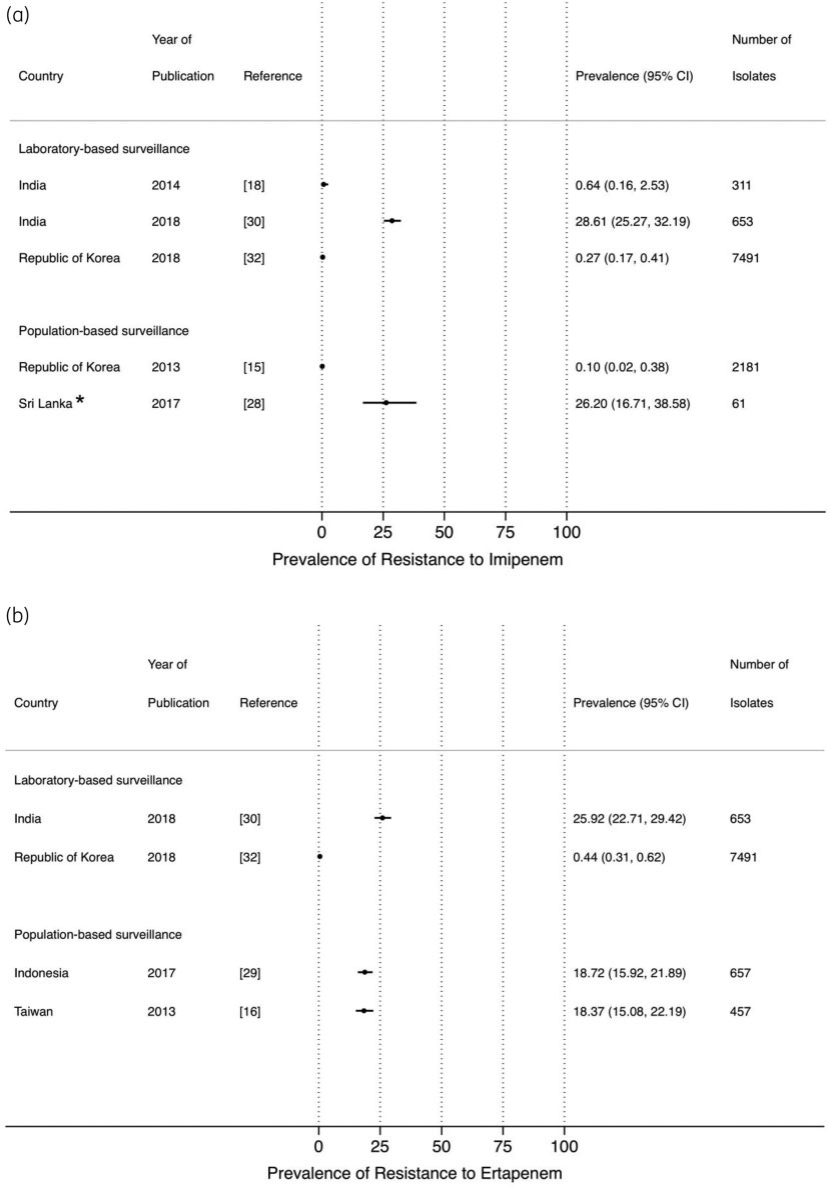
Point prevalence estimates of resistance against carbapenems in *E. coli* and *K. pneumoniae* by country in the Asia-Pacific region, stratified by surveillance strategy (laboratory-based surveillance, population-based surveillance): (a) imipenem, (b) ertapenem, (c) meropenem. The horizontal line indicates the 95% CI. An asterisk (*) indicates the study only included ESBL isolates.

**Figure 7.**
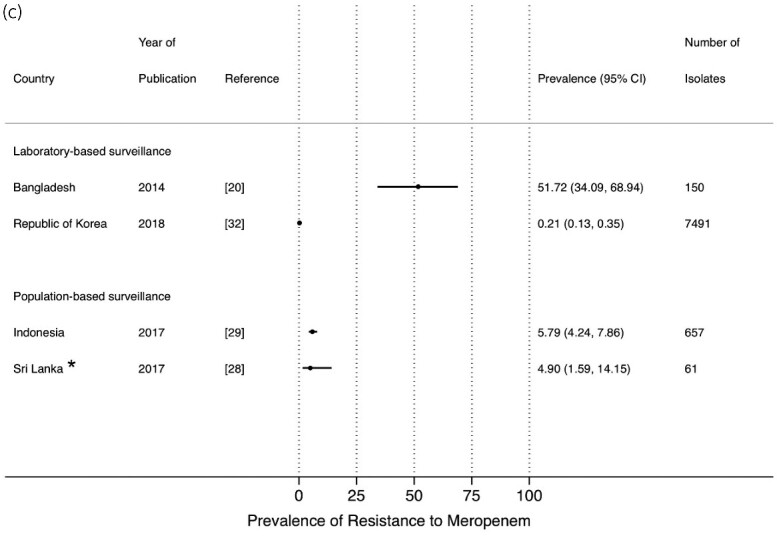
Continued.

## Discussion

We performed a systematic review of studies reporting on the prevalence of AMR in urinary pathogens in the Asia-Pacific region, with a focus on *E. coli* and *K. pneumoniae*, according to WHO recommendations.^2^ We observed high AMR prevalence estimates across the different classes of antimicrobial drugs that are used for the treatment of UTIs, except for fosfomycin. Ciprofloxacin and co-trimoxazole were the antimicrobial drugs most commonly reported, reflecting their frequent use as first line and empirical treatment in most hospital and/or community settings for treating UTIs.^4^^,^^5^^,^^34^ However, our systematic search, which targeted 41 countries over a 12 year period, only yielded 24 studies that fulfilled the inclusion criteria. We have previously noted a similar paucity of surveillance studies on AMR in UTIs in a systematic review on AMR in clinical syndromes in the African region.^36^ As noted then, the paucity of data is surprising given that urine culture is relatively easy to perform, both with respect to obtaining a sample and to laboratory requirements, and AMR in urinary pathogens from out-patients can give an indication of resistance prevalence in the community in general. We included studies from 11 countries in the APAC region, with a limited number of countries contributing the majority of studies, including India, Bangladesh, Sri Lanka, Thailand, Indonesia, Australia and Republic of Korea. These data suggest that in the majority of APAC countries, patients receive empirical treatment of UTIs in the absence of published national surveillance data to inform treatment strategies.

We reported the results for *E. coli* and *K. pneumoniae* combined as this is most informative for empirical treatment, given the predominant contribution of these two pathogens to UTI. We included studies that performed LBS and PBS and which included a range of patient populations, including in- and outpatients, patients with urinary catheters, and patients with lower and upper, and complicated and uncomplicated UTIs (Table S1) with risk of bias, as previously reported.^37–40^ However, whilst the data represent two bacterial species isolated from urinary samples only, the high prevalence estimates reported from India, Bangladesh and Sri Lanka are consistent with the high prevalence of resistance reported from a range of surveillance studies and programmes addressing other clinical syndromes and pathogens from these countries.^4^^,^^41^^,^^42^

We assessed the quality of articles and found only a limited number of studies that reported on the presence of quality control procedures for AST. Although most of the laboratories reported the guidelines used for susceptibility testing and breakpoints, variation in quality of identification and susceptibility testing may contribute to variation in prevalence estimates.^43^ In addition, inconsistent reporting of susceptibility test results precluded an analysis by susceptible, intermediate and resistant test result categories, which may have inflated resistance prevalence estimates because, if intermediate test results were available, intermediate susceptible test results were classified as resistant. These results further reflect the need for standardized and quality laboratory procedures and reporting.^44^

Our review has several limitations. First, we were unable to retrieve some articles, despite our access to major online database and medical libraries. In addition, we reported data from community-based and hospital-associated as well as in- and out-patients combined, for both laboratory-based and population-based surveillance of UTIs. Second, we found heterogeneity across the studies which precluded a meaningful analysis of trends over time, even within countries. For example, five studies from India each reported data from different populations, i.e. from adult female patients in the out-patient department (OPD); from in-patients; from male and female patients attending OPD; from paediatric in- and out-patients, and for Gram-negative isolates from unspecified populations (Table S1). Furthermore, there is variation in the antibiotics included in the studies. Therefore, it would not be possible to interpret the results of a combined analysis of trends over time. For the same reason, a direct comparison between LBS and PBS data is not possible, although Figures 2 and 4–7 suggest that LBS prevalence estimates are higher than PBS. However, in a direct comparison in a study in Indonesia^40^ it was demonstrated that LBS estimates indeed tend to be biased towards higher prevalence of resistance compared with PBS.

In conclusion, the results of our systematic review show high prevalence among uropathogens of AMR to co-trimoxazole and ciprofloxacin, which are commonly used for the empirical treatment of UTI, in most of the countries in the APAC for which data are available. The main treatment option appears to be fosfomycin as recommended in several guidelines, which is not always accessible for patients.^34^^,^^35^ The high prevalence estimates of AMR are observed in countries which have only recently developed or implemented their national surveillance system, such as Indonesia, and Bangladesh and India, respectively. For most other countries in the region, AMR surveillance data on urinary pathogens are still lacking. Clearly, whilst AMR surveillance is crucial to understand the burden of AMR and to inform empirical treatment, novel feasible and affordable approaches that facilitate population-based AMR surveillance, such as previously reported,^7^ are needed to provide reproducible local AMR prevalence estimates to inform empirical treatment guidelines and to contribute to national surveillance data to monitor trends.

## Funding

This work was supported by grant of the Royal Netherlands Academy of Arts and Sciences as part of the Scientific Program Indonesia-the Netherlands (SPIN) (project number: SPIN3-JRP-30).

## Transparency declarations

None to declare.

### Author contributions

A.K.S. and C.S. conceived the study. A.K.S., C.S. and F.vL. designed the study. A.K.S. and F.G. searched published work, reviewed published papers and made the primary selection of eligible papers. C.S. and F.vL. resolved disagreements regarding the eligibility of papers. A.K.S. and C.S. compiled the data. A.K.S., C.S. and F.vL. analysed the data. All authors contributed to the writing of the report and have seen and approved the final version.

## Supplementary data

Supplementary Methods and Tables S1 to S4 are available as Supplementary data at *JAC-AMR* Online.

## Supplementary Material

dlab003_Supplementary_Data
