# Supplementary material for: Antimicrobial resistance among uropathogens in the Asia-Pacific region: a systematic review
Source: JAC Antimicrob Resist. 2021 Feb 27;3(1):dlab003. doi: 10.1093/jacamr/dlab003 (PMC8210283; doi:10.1093/jacamr/dlab003)
Supplement: dlab003_Supplementary_Data [file dlab003_supplementary_data.docx]

**Supplementary data**

Supplementary Methods 1

**Search Strategy**

The search was carried out initially in June 2018 and was updated in January 2020 (see #6).

| Search Sequence | Search Strategy |
| --- | --- |
| #1 | antimicrobial resistan*[Title/Abstract] OR antibiotic resistan*[Title/Abstract] OR antibiotics resistan*[Title/Abstract] OR antimicrobial drug resistan*[Title/Abstract] OR antimicrobials drug resistan*[Title/Abstract] OR antimicrobial drugs resistan*[Title/Abstract] OR antimicrobials drugs resistan*[Title/Abstract] OR antimicrobials resistan*[Title/Abstract] |
| #2 | surveillan* OR bacterial surveillan* OR bacterials surveillan* OR epidemiology OR prevalence* OR incidence* |
| #3 | survei* OR bacterial survei* OR bacterials survei* OR epidemiology OR prevalence* OR incidence* |
| #4 | survey* OR bacterial survey* OR bacterials survey* OR epidemiology OR prevalence* OR incidence* |
| #5 | urinary tract infection OR urinary tract infection* OR UTI* OR uropathogen* |
| #6 | 2008[Date - Publication] /2018/07/01""[Date - Publication]: 3000[Date - Publication] AND Human*[MeSH Terms] |

Supplementary Methods 2

**Modified Newcastle-Ottawa Scale for Quality Assessment of cohort studies**

Definition of Population

(1). Is the study population clearly described (e.g. type of UTI, age group)? (Y/N)

(2). Are the criteria (case definition) for enrolment in the study clearly stated? (Y/N)

Representativeness of the sample

(3). Is the sampling of target population clearly described? (Y/N)

Ascertainment of AST Method

(4). Does the study describe the type of susceptibility testing used? (Y/N)

(5). Did the study specify the testing standard used (e.g. CLSI / EUCAST breakpoints)? (Y/N)

(6). Did the study describe any internal quality control measures (e.g. use of test control strains)? (Y/N)

abbrev: Y, Yes; N, No

Table S1. **Characteristics of studies included in review**

| **Reference** | **Author** | **Year**  **Published** | **Country Reported** | **Surveillance Type** | **Study Type** | **Population** | **Number of isolates**§  **(*Eco/Kpn**)** | **Microorganism identification** | **Susceptibility Testing** | **Breakpoint Guideline** | **Quality**  **Control** |
| --- | --- | --- | --- | --- | --- | --- | --- | --- | --- | --- | --- |
| 10 | Kothari | 2008 | India | Laboratory | Prospective | Adult, non-pregnant females in the outpatient department of 5 different hospital; had clinical evidence of a urinary tract infection, as determined by the treating physician | 361  (361/0) | API | Disk Diffusion | CLSI | Yes |
| 11 | Kim | 2008 | Korea | Population | Prospective | Women who had acute uncomplicated cystitis, who presented to outpatient urology clinics | 214  (214/0) | Unknown | Vitek | CLSI | No |
| 12 | Ho | 2010 | Hong Kong | Population | Prospective | Adult women (aged ≥18 years) with outpatient diagnosis of uncomplicated cystitis from general practitioners' (GP) offices, general out- patient clinics (GOPCs), and emergency departments (EDs) | 271  (271/0) | Vitek | Disk Diffusion | CLSI | Yes |
| 13 | Lee SJ | 2011 | Korea | Population | Prospective | Any adult patient with clinical and microbiological diagnosis of UTI | 1494  (1414/80) | Microscan | MIC Dilution | Unknown | No |
| 14 | Lu PL | 2012 | Asia Pacific | Laboratory | Prospective | Laboratory isolate from patients with upper UTIs, collected from participating hospitals among 10 countries | 1238  (995/243) | Unknown | Microscan | CLSI | Yes |
| 15 | Lee DS | 2013 | Korea | Population | Prospective | Any female patient between 25 and 65 years of age who had a clinical and microbiological UTI diagnosis from inpatient and outpatient. | 2181  (1991/190) | Microscan | MIC Dilution | CLSI | No |
| 16 | Chen | 2013 | Taiwan | Population | Retrospective | Hospitalized adults identified by a discharge diagnosis of UTI in hospital | 457  (416/41) | Unknown | Phoenix | CLSI | Unknown |
| 17 | Mitchell | 2014 | Australia | Laboratory | Prospective | Isolates were collected from non-hospitalized patients with urinary tract infections (emergency departments, outpatient departments or to community practitioners) | 2459 (2025/434) | Combination | Vitek | CLSI | Yes |
| 18 | Niranjan | 2014 | India | Laboratory | Prospective | All E. coli isolates obtained from urine samples among hospital inpatient with UTI | 311  (311/0) | Unknown | Disk Diffusion | CLSI | No |
| 19 | Kapur | 2014 | India | Laboratory | Retrospective | All pathogens isolated from urine specimens of patients (both male and female; age 14-72 y) who attended the outpatient departments (OPDs) in hospital and local diagnostic laboratories, with clinical suspicion of UTI. | 201  (161/40) | Chromogenic Agar | Disk Diffusion | CLSI | No |
| 20 | Hossain | 2014 | Bangladesh | Laboratory | Prospective | Hospitalized non-catheterized patients with UTI and catheterized patients with UTI | 150  (149/1) | Biochemical | Disk Diffusion | CLSI | Unknown |
| 21 | Senadheera | 2016 | Sri Lanka | Population | Prospective | Patients who attended the outpatient departments (OPD) or medical clinics of these hospitals with symptoms suggestive of UTI, and requested urine culture and AST. | 204  (204/0) | Chromogenic Agar | Disk Diffusion | CLSI | Unknown |
| 22 | Fasugba | 2016 | Australia | Laboratory | Retrospective | Positive E. coli urine cultures from community and hospital-acquired UTIs | 5333  (5333/0) | Unknown | Vitek | CLSI | Yes |
| 23 | Amornchaicharoensuk | 2016 | Thailand | Population | Prospective | Patient admitted to Hospital, diagnosed with UTI | 88  (88/0) | Unknown | Unknown | Unknown | Unknown |
| 24 | Jean | 2016 | Asia Pacific | Laboratory | Prospective | Laboratory isolate of patients with UTIs from Thirteen Asia-Pacific countries | 6992  (5689/1303) | Unknown | MIC Dilution | CLSI | Yes |
| 25 | Adeep | 2016 | Bhutan | Laboratory | Prospective | Urine culture done in the microbiology laboratory, from outpatient, assuming for simple /uncomplicated symptomatic cystitis. | 624  (594/30) | Unknown | Disk Diffusion | CLSI | Yes |
| 26 | Mishra | 2016 | India | Laboratory | Prospective | Pathogenic isolate isolated from urine samples of OPD and IPD pediatric patients with complaints of fever and foul urine | 154  (109/45) | Chromogenic Agar | Disk Diffusion | CLSI | No |
| 27 | Pruetpongpun | 2017 | Thailand | Population | Prospective | Patients with Acute Uncomplicated Cystitis presenting at the GP outpatient clinic | 47  (47/0) | Unknown | Disk Diffusion | CLSI | Unknown |
| 28 | Fernando | 2017 | Sri Lanka | Population | Prospective | Consecutive adult patient who had a culture positive UTI caused by ESBL producers. | 61  (53/8) | Unknown | Disk Diffusion | CLSI | Unknown |
| 29 | Sugianli | 2017 | Indonesia | Population | Prospective | Patient with lower or upper UTI from Inpatient and outpatient | 657  (521/136) | Biochemical | Disk Diffusion | CLSI | Yes |
| 30 | Veeraraghavan | 2018 | India | Laboratory | Prospective | Clinical isolates of GNB isolated from IAIs, UTIs and RTIs, across different hospitals | 653 (465/188) | Biochemical | Microscan | CLSI | Yes |
| 31 | Choe HS | 2018 | Asia Pacific | Population | Prospective | Medical record of Asian countries; collected to determine antibiotic use in urology departments and susceptibility of uropathogens causing UTIs | 255  (255/0) | Unknown | Unknown | Unknown | Unknown |
| 32 | Lee H | 2018 | Korea | Laboratory | Prospective | Clinical isolates collected from six sentinel hospitals according to GLASS | 7491  (6394/1097) | Combination | Combination | CLSI | No |
| 33 | Ganesh | 2019 | Nepal | Population | Prospective | IPD and OPD Pediatric patient with complaints of fever and foul urine | 159  (114/45) | Biochemical | Disk Diffusion | CLSI | Yes |

(*) Eco: *Escherichia coli*; Kpn: *Klebsiella pneumoniae*

(§) Number of isolates used for the analysis may differ from the total number of isolates in the original paper, because only included *Escherichia coli* and *Klebsiella pneumoniae*

Table S2. **Characteristics of data reported of studies included in review**

| **Ref** | **Author** | **Year** | **Country** | **Data Reported** | **Remark** | **Estimate Calculation for This Study** |
| --- | --- | --- | --- | --- | --- | --- |
| 10 | Kothari | 2008 | India | %S | No information about intermediate category | 100 - %S |
| 11 | Kim | 2008 | Korea | %S | No information about intermediate category | 100 - %S |
| 12 | Ho | 2010 | Hong Kong | %S | No information about intermediate category | 100 - %S |
| 13 | Lee SJ | 2011 | Korea | %R | No information about intermediate category | %R |
| 14 | Lu PL | 2012 | Asia Pacific | %S | No information about intermediate category | 100 - %S |
| 15 | Lee DS | 2013 | Korea | %S, %I, %R | %S, %I, %R reported separately | %R |
| 16 | Chen | 2013 | Taiwan | %S | No information about intermediate category | 100 - %S |
| 17 | Mitchell | 2014 | Australia | %R, %NS | Intermediate was categorized as non-susceptible | %NS for antibiotic with the intermediate category  %R for antibiotic without intermediate category |
| 18 | Niranjan | 2014 | India | %R | No information about intermediate category | %R |
| 19 | Kapur | 2014 | India | %S, %I, %R | %S, %I, %R reported separately | %R |
| 20 | Hossain | 2014 | Bangladesh | %R | No information about intermediate category | %R |
| 21 | Senadheera | 2016 | Sri Lanka | %R | No information about intermediate category | %R |
| 22 | Fasugba | 2016 | Australia | %R | No information about intermediate category | %R |
| 23 | Amornchaicharoensuk | 2016 | Thailand | %R | No information about intermediate category | %R |
| 24 | Jean | 2016 | Asia Pacific | %S | No information about intermediate category | %R |
| 25 | Adeep | 2016 | Bhutan | %R | No information about intermediate category | %R |
| 26 | Mishra | 2016 | India | %R | No information about intermediate category | %R |
| 27 | Pruetpongpun | 2017 | Thailand | %R | No information about intermediate category | %R |
| 28 | Fernando | 2017 | Sri Lanka | %S, %R | %S, %R reported separately  No information about intermediate category | %R |
| 29 | Sugianli | 2017 | Indonesia | %R | Intermediate consider as resistant | %R |
| 30 | Veeraraghavan | 2018 | India | %S | No information about intermediate category | 100 - %S |
| 31 | Choe HS | 2018 | Asia Pacific | %R | No information about intermediate category | %R |
| 32 | Lee H | 2018 | Korea | %S, %I, %R | %S, %I, %R reported separately | %R |
| 33 | Ganesh | 2019 | Nepal | %R | No information about intermediate category | %R |

abbrev: %S, percentage of susceptible; %I, percentage of intermediate; %R, percentage of resistant; %NS, percentage of non-susceptible

Table S3. **Total number of study isolates and number of intermediate/resistant isolates, stratified by Laboratory-based and Population-based surveillance**

| **Laboratory-based Surveillance** | | | | | | | | | | | | | | | |
| --- | --- | --- | --- | --- | --- | --- | --- | --- | --- | --- | --- | --- | --- | --- | --- |
| **Country** | **Author** | **Year of Publication** | **N**^§^ | **SXT** | | **CIP** | | **LVX** | | **CRO** | | **CTX** | | **CAZ** | |
|  |  |  |  | **n** | **nr*** | **n** | **nr*** | **n** | **nr*** | **n** | **nr*** | **n** | **nr*** | **n** | **nr*** |
| Australia | Mitchell | 2014 | 2459 | 2459 | 503 | 2459 | 155 | - | - | 2459 | 105 | - | - | 2459 | 58 |
| Australia | Fasugba | 2016 | 5333 | 5326 | 1102 | 4407 | 286 | - | - | - | - | - | - | - | - |
| Bangladesh | Hossain | 2014 | 150 | 150 | 87 | 142 | 78 | - | - | 149 | 92 | - | - | 148 | 98 |
| Bhutan | Adeep | 2016 | 624 | 624 | 330 | - | - | - | - | 624 | 126 | - | - | - | - |
| India | Kothari | 2008 | 361 | 361 | 267 | 361 | 260 | - | - | - | - | - | - | - | - |
| India | Niranjan | 2014 | 311 | 311 | 200 | 311 | 233 | - | - | - | - | - | - | - | - |
| India | Kapur | 2014 | 201 | - | - | 201 | 10/150 | - | - | - | - | - | - | - | - |
| India | Mishra | 2016 | 154 | 154 | 51 | - | - | 154 | 54 | 154 | 51 | - | - | 154 | 48 |
| India | Veeraraghavan | 2018 | 653 | - | - | 653 | 506 | 653 | 465 | 653 | 488 | 653 | 491 | 653 | 447 |
| Republic of Korea | Lee H | 2018 | 7491 | 7491 | 153/2775 | 7491 | 96/3235 | - | - | - | - | 7491 | 100/2433 | 7491 | 506/984 |
|  |  |  |  |  |  |  |  |  |  |  |  |  |  |  |  |
| **Country** | Author | **Year of Publication** | **N**^§^ | **FEP** | | **IMP** | | **MEM** | | **ETP** | | **FOS** | | **NIT** | |
|  |  |  |  | **n** | **nr*** | **n** | **nr*** | **n** | **nr*** | **n** | **nr*** | **n** | **nr*** | **n** | **nr*** |
| Australia | Mitchell | 2014 | 2459 | 2459 | 16 | - | - | 2459 | 0 | - | - | - | - | 2035 | 110 |
| Australia | Fasugba | 2016 | 5333 | - | - | - | - | - | - | - | - | - | - | 5333 | 144 |
| Bangladesh | Hossain | 2014 | 150 | - | - | - | - | 29 | 15 | - | - | - | - | - | - |
| Bhutan | Adeep | 2016 | 624 | - | - | - | - | - | - | - | - | - | - | 624 | 18 |
| India | Kothari | 2008 | 361 | - | - | - | - | 361 | 0 | - | - | - | - | 361 | 88 |
| India | Niranjan | 2014 | 311 | - | - | 311 | 3 | - | - | - | - | - | - | 311 | 56 |
| India | Kapur | 2014 | 201 | - | - | - | - | - | - | - | - | - | - | - | - |
| India | Mishra | 2016 | 154 | - | - | - | - | - | - | - | - | - | - | 154 | 45 |
| India | Veeraraghavan | 2018 | 653 | 653 | 470 | 653 | 187 | - | - | 653 | 169 | - | - | - | - |
| Republic of Korea | Lee H | 2018 | 7491 | 7491 | 775/1446 | 7491 | 8/12 | 7491 | 4/12 | 7491 | 13/20 | - | - | - | - |

abbrev: N, number of isolates; n, number of tested isolates; nr, number of resistance isolates; (-), not tested/reported; SXT, co-trimoxazole; CIP, ciprofloxacin; LVX, levofloxacin; CRO, ceftriaxone; CTX, cefotaxime; CAZ, ceftazidime; FEP, cefepime (FEP); IMP, imipenem; MEM, meropenem; ETP, ertapenem; FOS, fosfomycin; F, nitrofurantoin. (*) intermediate susceptible/resistant number of isolates, if applicable; (§) Number of isolates used for the analysis may differ from the total number of isolates in the original paper, because only included *Escherichia coli* and *Klebsiella pneumoniae.*

| **Population-based Surveillance** | | | | | | | | | | | | | | | |
| --- | --- | --- | --- | --- | --- | --- | --- | --- | --- | --- | --- | --- | --- | --- | --- |
| **Country** | **Author** | **Year of Publication** | **N**^§^ | **SXT** | | **CIP** | | **LVX** | | **CRO** | | **CTX** | | **CAZ** | |
|  |  |  |  | **n** | **nr*** | **n** | **nr*** | **n** | **nr*** | **n** | **nr*** | **n** | **nr*** | **n** | **nr*** |
| Hong Kong | Ho | 2010 | 271 | 271 | 83 | 271 | 35 | - | - | - | - | - | - | - | - |
| Indonesia | Sugianli | 2017 | 657 | 657 | 445 | 657 | 500 | 657 | 470 | 657 | 475 | - | - | 657 | 462 |
| Nepal | Ganesh | 2019 | 159 | 159 | 79 | - | - | - | - | - | - | 159 | 67 | - | - |
| Republic of Korea | Kim | 2008 | 214 | 214 | 63 | 214 | 50 | - | - | - | - | - | - | - | - |
| Republic of Korea | Lee SJ | 2011 | 1494 | 1494 | 477 | 1494 | 385 | 1494 | 335 | 1494 | 106 | - | - | - | - |
| Republic of Korea | Lee DS | 2013 | 2181 | 2084 | 0/732 | 2082 | 36/645 | - | - | - | - | 2078 | 4/316 | 2079 | 5/320 |
| Sri Lanka | Senadheera | 2016 | 204 | 204 | 96 | 199 | 92 | - | - | - | - | - | - | - | - |
| Sri Lanka^†^ | Fernando | 2017 | 61 | - | - | 61 | 55 | - | - | 61 | 61 | - | - | 61 | 61 |
| Taiwan | Chen | 2013 | 457 | 457 | 220 | - | - | 457 | 69 | 457 | 114 | - | - | - | - |
| Thailand | Amornchaicharoensuk | 2016 | 88 | 88 | 53 | 88 | 18 | - | - | 88 | 19 | 88 | 20 | 88 | 20 |
| Thailand | Pruetpongpun | 2017 | 48 | 47 | 29 | - | - | - | - | 47 | 10 | - | - | - | - |
|  |  |  |  |  |  |  |  |  |  |  |  |  |  |  |  |
| **Country** | **Author** | **Year of Publication** | **N**^§^ | **FEP** | | **IMP** | | **MEM** | | **ETP** | | **FOS**^‡^ | | **NIT** | |
|  |  |  |  | **n** | **nr*** | **n** | **nr*** | **n** | **nr*** | **n** | **nr*** | **n** | **nr*** | **n** | **nr*** |
| Hong Kong | Ho | 2010 | 271 | - | - | - | - | - | - | - | - | 271 | 5 | 271 | 21 |
| Indonesia | Sugianli | 2017 | 657 | 657 | 426 | - | - | 657 | 38 | 657 | 123 | 500 | 9 | 657 | 206 |
| Nepal | Ganesh | 2019 | 159 | - | - | - | - | - | - | - | - | - | - | 159 | 15 |
| Republic of Korea | Kim | 2008 | 214 | - | - | - | - | - | - | - | - | - | - | - | - |
| Republic of Korea | Lee SJ | 2011 | 1494 | - | - | - | - | - | - | - | - | - | - | - | - |
| Republic of Korea | Lee DS | 2013 | 2181 | 2079 | 1/314 | 2085 | 0/2 | - | - | - | - | - | - | - | - |
| Sri Lanka | Senadheera | 2016 | 204 | - | - | - | - | - | - | - | - | - | - | 202 | 30 |
| Sri Lanka^†^ | Fernando | 2017 | 61 | - | - | 61 | 16 | 61 | 3 | - | - | - | - | 61 | 33 |
| Taiwan | Chen | 2013 | 457 | - | - | 457 | 0 | - | - | 457 | 84 | - | - | - | - |
| Thailand | Amornchaicharoensuk | 2016 | 88 | 88 | 19 | - | - | - | - | - | - | - | - | - | - |
| Thailand | Pruetpongpun | 2017 | 48 | - | - | - | - | - | - | 47 | 0 | - | - | - | - |

abbrev: N, number of isolates; n, number of tested isolates; nr, number of resistance isolates; dash ( - ), not tested/reported; SXT, co-trimoxazole; CIP, ciprofloxacin; LVX, levofloxacin; CRO, ceftriaxone; CTX, cefotaxime; CAZ, ceftazidime; FEP, cefepime (FEP); IMP, imipenem; MEM, meropenem; ETP, ertapenem; FOS, fosfomycin; F, nitrofurantoin. (*) intermediate susceptible/resistant number of isolates, if applicable; (†) This study only included ESBL isolates; (§) Number of isolates used for the analysis may differ from the total number of isolates in the original paper, because only included *Escherichia coli* and *Klebsiella pneumoniae*; (‡) Only tested for *Escherichia coli.*

Table S4. **Total number of study isolates and number of intermediate/resistant isolates in studies aggregating data from multiple countries**

| **Author** | **Year of Publication** | **Surveillance** | **N**^§^ | **SXT** | | **%R (95%CI)** | **CIP** | | **%R (95%CI)** | **LVX** | | **%R (95%CI)** |
| --- | --- | --- | --- | --- | --- | --- | --- | --- | --- | --- | --- | --- |
|  |  |  |  | **n** | **nr*** |  | **n** | **nr*** |  | **n** | **nr*** |  |
| Lu PL | 2012 | LBS | 1238 | - | - | - | 1238 | 646 | 52.19(49.40-54.96) | 1238 | 619 | 50.01(47.23-52.79) |
| Jean | 2016 | LBS | 6992 | - | - | - | 6992 | 3559 | 50.90(49.73-52.07) | 6992 | 3311 | 47.35(46.18-48.52) |
| Choe HS | 2018 | PBS | 255 | 81 | 39 | 48.15(37.52-58.95) | 137 | 65 | 47.45(39.23-55.80) | - | - | - |
|  |  |  |  |  |  |  |  |  |  |  |  |  |
| **Author** | **Year of Publication** | **Surveillance** | **N**^§^ | **CRO** | | **%R (95%CI)** | **CTX** | | **%R (95%CI)** | **CAZ** | | **%R (95%CI)** |
|  |  |  |  | **n** | **nr*** |  | **n** | **nr*** |  | **n** | **nr*** |  |
| Lu PL | 2012 | LBS | 1238 | 1238 | 553 | 44.64(41.89-47.42) | 1238 | 552 | 44.56(41.81-47.34) | 1238 | 393 | 31.77(29.94-34.42) |
| Jean | 2016 | LBS | 6992 | 6992 | 3086 | 44.13(42.97-45.30) | 6992 | 3119 | 44.61(43.44-45.77) | 6992 | 2235 | 31.96(30.88-33.06) |
| Choe HS | 2018 | PBS | 255 | - | - | - | 139 | 45 | 32.37(25.13-40.58) | - | - | - |

| **Author** | **Year of Publication** | **Surveillance** | **N**^§^ | **FEP** | | **%R (95%CI)** | **IMP** | | **%R (95%CI)** | **ETP** | | **%R (95%CI)** |
| --- | --- | --- | --- | --- | --- | --- | --- | --- | --- | --- | --- | --- |
|  |  |  |  | **n** | **nr*** |  | **n** | **nr*** |  | **n** | **nr*** |  |
| Lu PL | 2012 | LBS | 1238 | 1238 | 455 | 36.76(34.12-39.48) | 1238 | 9 | 0.73(0.38-1.39) | 1238 | 17 | 1.37(0.85-2.19) |
| Jean | 2016 | LBS | 6992 | 6992 | 2702 | 38.65(37.51-39.79) | 6992 | 88 | 1.26(1.02-1.55) | 6992 | 160 | 2.29(1.96-2.66) |
| Choe HS | 2018 | PBS | 255 | - | - | - | - | - | - | - | - | - |

abbrev: N, number of isolates; n, number of tested isolates; nr, number of resistance isolates; (-), not tested/reported; 95%CI, 95% Confidence Interval; SXT, co-trimoxazole; CIP, ciprofloxacin; LVX, levofloxacin; CRO, ceftriaxone; CTX, cefotaxime; CAZ, ceftazidime; FEP, cefepime (FEP); IMP, imipenem; MEM, meropenem; ETP, ertapenem; FOS, fosfomycin; F, nitrofurantoin; LBS, Laboratory-based surveillance; PBS, Population-based surveillance; %R, point prevalence estimates of resistance; (*) intermediate susceptible/resistant number of isolates, if applicable; (§) Number of isolates used for the analysis may differ from the total number of isolates in the original paper, because only included *Escherichia coli* and *Klebsiella pneumoniae.*
